# Preoperative prediction of lymphovascular invasion in patients with T1 breast invasive ductal carcinoma based on radiomics nomogram using grayscale ultrasound

**DOI:** 10.3389/fonc.2022.1071677

**Published:** 2022-12-07

**Authors:** Mao-Lin Xu, Shu-E Zeng, Fang Li, Xin-Wu Cui, Gui-Feng Liu

**Affiliations:** ^1^ Department of Radiology, China-Japan Union Hospital of Jilin University, Changchun, China; ^2^ Department of Ultrasound, Hubei Cancer Hospital, Tongji Medical College, Huazhong University of Science and Technology, Wuhan, China; ^3^ Department of Medical Ultrasound, Tongji Hospital, Tongji Medical College, Huazhong University of Science and Technology, Wuhan, China

**Keywords:** lymphovascular invasion, breast invasive ductal carcinoma, ultrasound, radiomics, nomogram

## Abstract

**Purpose:**

The aim of this study was to develop a radiomics nomogram based on grayscale ultrasound (US) for preoperatively predicting Lymphovascular invasion (LVI) in patients with pathologically confirmed T1 (pT1) breast invasive ductal carcinoma (IDC).

**Methods:**

One hundred and ninety-two patients with pT1 IDC between September 2020 and August 2022 were analyzed retrospectively. Study population was randomly divided in a 7: 3 ratio into a training dataset of 134 patients (37 patients with LVI-positive) and a validation dataset of 58 patients (19 patients with LVI-positive). Clinical information and conventional US (CUS) features (called clinic_CUS features) were recorded and evaluated to predict LVI. In the training dataset, independent predictors of clinic_CUS features were obtained by univariate and multivariate logistic regression analyses and incorporated into a clinic_CUS prediction model. In addition, radiomics features were extracted from the grayscale US images, and the radiomics score (Radscore) was constructed after radiomics feature selection. Subsequent multivariate logistic regression analysis was also performed for Radscore and the independent predictors of clinic_CUS features, and a radiomics nomogram was developed. The performance of the nomogram model was evaluated *via* its discrimination, calibration, and clinical usefulness.

**Results:**

The US reported axillary lymph node metastasis (LNM) (US_LNM) status and tumor margin were determined as independent risk factors, which were combined for the construction of clinic_CUS prediction model for LVI in pT1 IDC. Moreover, tumor margin, US_LNM status and Radscore were independent predictors, incorporated as the radiomics nomogram model, which achieved a superior discrimination to the clinic_CUS model in the training dataset (AUC: 0.849 vs. 0.747; P < 0.001) and validation dataset (AUC: 0.854 vs. 0.713; P = 0.001). Calibration curve for the radiomic nomogram showed good concordance between predicted and actual probability. Furthermore, decision curve analysis (DCA) confirmed that the radiomics nomogram had higher clinical net benefit than the clinic_CUS model.

**Conclusion:**

The US-based radiomics nomogram, incorporating tumor margin, US_LNM status and Radscore, showed a satisfactory preoperative prediction of LVI in pT1 IDC patients.

## 1 Introduction

Lymphovascular invasion (LVI) refers to the presence of tumor cells in the lymphatic or vascular system surrounding invasive breast cancer (1, 2). LVI is positively associated with increased risk of axillary lymph node (ALN), distant metastasis, less responsive to chemotherapy, and locoregional recurrence (1, 3). Therefore, LVI has been considered as a major criteria for tumor staging, prognostic prediction, and the treatment choice (3, 4). Treatment decisions for T1 breast cancer usually need refer to the presence or absence of lymph node metastasis (5), which is often associated with LVI (1, 5–9). LVI had been proved as an independent prognostic factor in T1 breast cancer (9, 10), and patients with LVI may require more clinical decisions, including surgical type, the margin determination of surgery, and guiding more aggressive neoadjuvant treatment protocols for breast cancer patients (9, 11).

Currently, the presence of LVI is determined by postoperative pathology based on the primary tumor and peritumoral breast tissue (12, 13). However, several factors, such as partial sampling of tumors in preoperative biopsies, contractility of materials, and mechanical force-induced cell displacement, may complicate the postoperative assessment of LVI and lead to misdiagnosis (3, 14). Furthermore, preoperative neoadjuvant chemotherapy for breast cancer patients also makes it difficult to accurately evaluate LVI on the basis of postoperative specimens (3, 14).

Although preoperative biopsy can provide information about the histological type and immunohistochemistry of the tumor, biopsy is invasive and difficult to confirm LVI due to the small size of the tissue to be cut (12, 13). Therefore, it is necessary to find a simple, accurate and non-invasive method for preoperative prediction of tumor LVI, which would be of great significance for clinical decision-making in breast cancer (12).

Previous studies have demonstrated that preoperative breast mammography, digital breast tomosynthesis (DBT) or MRI can be effectively used for the prediction of LVI (1, 3, 6, 7, 15). Different from other imaging methods, US has the advantages of relatively low price, real-time, and reproducible operation, and is a reliable examination method for breast tumors in clinical work (16). Some CUS features had been demonstrated as the independent variables for predicting the presence of LVI in invasive breast cancer (8). Moreover, multiparametric ultrasound, especially contrast-enhanced ultrasound (CEUS), could also provide good discriminative value for predicting LVI (12). However, the cost of additional contrast-enhancement is higher and the examination time is longer (16). Additionally, the assessment of CUS features and the quantitative indicators of CEUS may be susceptible to subjective factors (12, 16).

Radiomics is a relatively new machine learning approach that provides high-throughput quantitative information on tumor shape, intensity, and texture (3, 6), which fail to be detected by naked eyes (17). Advances in radiomics-based US have increasingly highlighted its potential value for improving diagnosis, evaluating prognosis, and predicting response to treatment in breast carcinoma (18–21). As a tool to determine the appropriate treatment for patients, nomogram was developed based on comprehensive data to allow the clinician to assess the associated clinical risk more accurately (18, 22). The nomogram incorporating US radiomics score had showed potential diagnostic capabilities for triple-negative breast cancer and fibroadenoma (18). On the other hand, nomogram based on US radiomics analysis exhibited high accuracy in predicting axillary lymph node (ALN) tumor burden in breast cancer patients (21). To date, few studies about US-based radiomocs nomogram have addressed the prediction of LVI in breast cancer.

At present, due to the improvement of people’s health consciousness and the increase of breast cancer screening, the diagnosis rate of T1 breast cancer has increased (16, 23), in which the most common histological type is invasive ductal carcinoma (IDC) (16). Thus, the purpose of our study was to investigate whether a nomogram based on radiomics analysis of US imaging could predict the status of LVI in patients with pathologically confirmed T1 (pT1) IDC.

## 2 Methods

### 2.1 Patients

This retrospective study was approved by the ethics committee of the Hubei Cancer Hospital (No.: LLHBCH2021YN-001), and the requirement to obtain informed consent was waived. 643 female patients with 771 breast carcinomas at the Hubei Cancer Hospital (Wuhan, People’s Republic of China) between September 2020 and August 2022 were consecutive included in this study.

All patients underwent ultrasonic examination within 1 month before operation and were satisfied as the following inclusion criteria (1): breast carcinoma pathologically confirmed by the specimens obtained from surgical resection; (2) no biopsy or medical treatment before US examinations; (3) lesions with complete histopathological, immunohistochemical and US data. The exclusion criteria were as follows: (1) patients with distant metastases or a history of other malignancies (n = 35); (2) patients with bilateral breast tumors or ipsilateral multifocal tumors of the breast (n = 79); (3) patients with other histologic types or other stages of breast cancer (n = 326) (4) poor quality images (n = 11).

**Figure 1:**
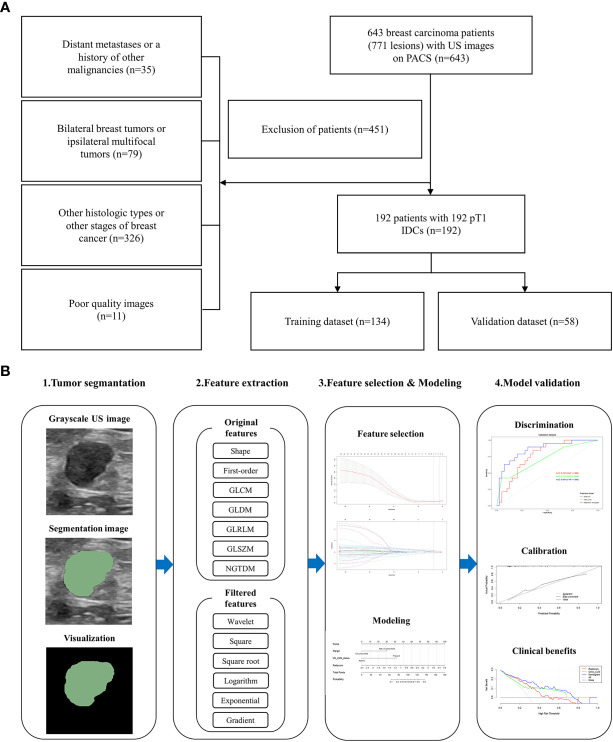
Flowchart of this study. **(A)** The patient selection process. **(B)** The workflow to construct and validate the radiomics nomogram.

Finally, 192 patients (mean age, 54.38 ± 11.27 years; range, 26 ~ 86 years) with 192 pT1 IDCs were eligible in our study, which were split into training and validation datasets with a 7:3 ratio by random sampling. The training and validation datasets included 134 patients and 58 patients, respectively. **Figure 1A** provides a flowchart of the patient selection process.

### 2.2 US examination

All patients underwent US examination using EPIQ5 (Philips Ultrasound, Inc., Bothell, Washington, USA; L12-5 linear-array transducer probe, 5 ~ 12 MHz), Aixplorer ultrasound scanner (SuperSonic Imagine, France; SL15-4 linear-array transducer probe, 4 ~ 15 MHz), and Resona 7, Resona 8, Resona R9, Resona I9, and DC-65 (Mindray, Shenzhen, China; L14-5, L14-3, and L13-3 linear-array transducer probes, 3 ~ 14 MHz). The image settings for each examination, including time gain compensation, focus position and dynamic range, were optimized according to the manufacturer’s recommendations.

After physical examination, the whole breast was systematically examined. All cases were evaluated in the supine position, with the upper arm abducted and the bilateral breast fully exposed at the same time. The characteristics of sonogram were observed by routine ultrasonic scanning. The US images were obtained in both longitudinal and transverse sections.

### 2.3 Clinical information and US conventional features

The clinical information, including serum CA153, CA125, and carcinoembryonic antigen (CEA) levels, and body mass index (BMI), were acquired by reviewing the medical records. BMI was calculated by dividing the patient’s weight (kg) by the square of the height (m^2^), with a cutoff value of 24 kg/m^2^ for classification (24).

All US images were reviewed and analyzed by two experienced radiologists (both with more than 5 years of breast image diagnosis experience). The two radiologists were blinded to the patient’s clinicopathological information. The clearest and most complete US images were obtained in DICOM format. The following conventional ultrasound (CUS) features of breast tumors were recorded in concordance with prior studies (12, 18): (1) tumor location: upper outer quadrant, upper inner quadrant, lower outer quadrant, lower inner quadrant or other positions; (2) tumor size: maximum diameter; (3) tumor shape: regular (round or oval) or irregular; (4) tumor margin: circumscribed or not circumscribed (indistinct, angular, microlobulated, or spiculated); (5) tumor orientation: parallel or not parallel; (6) tumor echo pattern: hypoechoic, isoechoic, hyperechoic or heterogeneous; (7) microcalcifications: present or absent; (8) tumor posterior features: no posterior acoustic features, enhancement, shadowing or combined pattern. In addition, suspicious CUS features of axillary lymph node metastasis (LNM) were also evaluated, including rounded hypoechoic node complete or partial effacement of the fatty hilum, the ratio of long axis diameter to short axis diameter < 2, cortical thickening > 3 mm, nonhilar cortical blood flow on color Doppler images, complete or partial replacement of the node with an ill-defined or irregular mass and microcalcifications in the node (25). With one or more of the above-mentioned suspicious features, the US reported LNM (US_LNM) status was recorded as present; otherwise, it was recorded as absent. Differences in interpretation of breast tumor features or US_LNM status between the two radiologists were determined by another radiologist with more than 10 years of experience in breast imaging diagnosis.

### 2.4 Histopathological analysis

In all 192 cases, specimens of breast cancer were surgically resected and the diagnosis were histopathologically confirmed. Nottingham combined histological grading system was used to determine the histological grade (26). The expression levels of estrogen receptor (ER), progesterone receptor (PR), human epidermal growth factor receptor 2 (HER-2), and Ki-67 antigen were measured through Immunohistochemical (IHC) analyses (16, 26). ≤1% of carcinoma nuclei with positive staining indicated that ER and PR were negative (16, 24, 26). The IHC score was - or 1 +, indicating that HER-2 was negative, while 3+ indicating the positive expression of HER-2. IHC score was 2 +, and fluorescence *in situ* hybridization (FISH) was negative, which also showed that HER-2 was negative (16, 26). The Ki-67 proliferation index was determined according to immunohistochemical analysis. An index of <14% was considered as low proliferation, while ≥14% is considered as high proliferation (12). According to previous study (27), patients were classified into the following four breast cancer subtypes: luminal A, luminal B, HER-2-positive, and triple-negative. LVI was defined as the presence of tumor cells within the lymphatic or vascular channels at the peritumoral region (6, 7), which was also identified by examining the primary tumor stained with endothelial-specific markers, including anti-CD34 and anti-D2-40 antibodies (6). The status of tumor LVI was defined as LVI-positive and LVI-negative.

### 2.5 Lesion segmentation and feature extraction in radiomics model

The ROI (region of interest) was manually delineated along the edge of the maximum diameter area for each tumor on grayscale US image. The work of Lesion segmentation was accomplished by Two radiologists (radiologist 1 and radiologist 2, both with more than 5 years of breast image diagnosis experience), who were blinded to the clinicopathological data.

Radiologist 1 drew all the ROIs by using 3D Slicer (version 4.11; http://www.slicer.org). To assess inter-observer reliability, radiologist 2 drew the ROIs in 40 randomly selected tumors. Additionally, to evaluate intra-observer reliability, Radiologist 1 then repeated the same procedure for second ROIs depiction from the 40 randomly selected images with an interval of 2 weeks period. Intraclass correlation coefficient (ICC) was then calculated to evaluate intra and inter-observer agreement for feature extraction. The intra and inter-observer ICC > 0.75 indicated good reproducibility of feature extraction.

Before feature extraction, image resampling and grayscale discretization were performed on the grayscale US image to achieve normalization (28–30): firstly, the images were resampled by B-spline interpolation to a pixel size of 1 mm × 1 mm; secondly, the gray-level discretization was performed in the histogram with a fixed bin width of 25. Then, each patient yielded 939 radiomics features as follows (1): two-dimensional (2D) shape-based features (n=9); (2) first-order features (n=18); (3) gray level co-occurrence matrix (GLCM) features (n=24); (4) gray-level dependence matrix (GLDM) features (n=14); (5) gray-level run length matrix (GLRLM) features (n=16); (6) gray-level size zone matrix (GLSZM) features (n=16); (7) neighboring gray tone difference matrix (NGTDM) features (n=5) and (8) transform-filtered features (including wavelet, square, square root, logarithm, exponential, gradient) (n= 837). (1) ~ (7) were the radiomics features from original images (**Table 1**), while (8) were were the radiomics features from derived images which were obtained by applying filters to the original image. The feature extraction was implemented by the open-source Pyradiomics package (version 3.0.1; https://pyradiomics.readthedocs.io/en/v3.0.1/)

**Table 1:** Original radiomics features extracted in current study.

Original radiomics features (n=102)	Feature names
Two-dimensional (2D) shape-based features (n=9)	Elongation; Major Axis Length; Maximum diameter; Mesh Surface; Minor Axis Length; Perimeter; Perimeter to Surface ratio; Pixel Surface; Sphericity
First-order statistics features (n=18)	Energy; Total Energy; Entropy; Minimum; 10th percentile; 90th percentile; Maximum; Mean; Median; Interquartile Range; Range; Mean Absolute Deviation (MAD); Robust Mean Absolute Deviation (rMAD); Root Mean Squared (RMS); Skewness; Kurtosis; Variance; Uniformity
Gray level co-occurrence matrix (GLCM) features (n=24)	Autocorrelation; Joint Average; Cluster Prominence; Cluster Shade; Cluster Tendency; Contrast; Correlation; Difference Average; Difference Entropy; Difference Variance; Joint Average; Joint Energy; Joint Entropy; Informational Measure of Correlation (IMC) 1; Informational Measure of Correlation (IMC) 2; Inverse Difference Moment (IDM); Inverse Difference Moment Normalized (IDMN); Inverse Difference (ID); Inverse Difference Normalized (IDN); Inverse Variance; Maximum Probability; Sum Average; Sum Entropy; Sum of Squares
Gray level dependence matrix (GLDM) features (n=14)	Dependence Entropy (DE); Dependence Non-Uniformity (DN); Dependence Non-Uniformity Normalized (DNN); Dependence Variance (DV); Gray Level Non-Uniformity (GLN); Gray Level Variance (GLV); High Gray Level Emphasis (HGLE); Large Dependence Emphasis (LDE); Large Dependence High Gray Level Emphasis (LDHGLE); Large Dependence Low Gray Level Emphasis (LDLGLE); Low Gray Level Emphasis (LGLE); Small Dependence Emphasis (SDE); Small Dependence High Gray Level Emphasis (SDHGLE); Small Dependence Low Gray Level Emphasis (SDLGLE)
Gray level run length matrix (GLRLM) features (n=16)	Gray Level Non-Uniformity (GLN); Gray Level Non-Uniformity Normalized (GLNN); Gray Level Variance (GLV); High Gray Level Run Emphasis (HGLRE); Long Run Emphasis (LRE); Long Run High Gray Level Emphasis (LRHGLE); Long Run Low Gray Level Emphasis (LRLGLE); Low Gray Level Run Emphasis (LGLRE); Run Entropy (RE); Run Length Non-Uniformity (RLN); Run Length Non-Uniformity Normalized (RLNN); Run Percentage (RP); Run Variance (RV); Short Run Emphasis (SRE); Short Run High Gray Level Emphasis (SRHGLE); Short Run Low Gray Level Emphasis (SRLGLE)
Gray level size zone matrix (GLSZM) features (n=16)	Gray Level Non-Uniformity (GLN); Gray Level Non-Uniformity Normalized (GLNN); Gray Level Variance (GLV); High Gray Level Zone Emphasis (HGLZE); Large Area Emphasis (LAE); Large Area High Gray Level Emphasis (LAHGLE); Large Area Low Gray Level Emphasis (LALGLE); Low Gray Level Zone Emphasis (LGLZE); Size-Zone Non-Uniformity (SZN); Size-Zone Non-Uniformity Normalized (SZNN); Small Area Emphasis (SAE); Small Area High Gray Level Emphasis (SAHGLE); Small Area Low Gray Level Emphasis (SALGLE); Zone Entropy (ZE); Zone Percentage (ZP); Zone Variance (ZV)
Neighborhood gray tone difference matrix (NGTDM) features (n=5)	Busyness; Coarseness; Complexity; Contrast; Strength

### 2.6 Feature selection, Radscore establishment, and nomogram construction

#### 2.6.1 Feature selection and Radscore establishment

Because of the poor reproducibility, the radiomics features with intra-ICC or inter-ICC ≤ 0.75 were initially excluded (31). Then, a training/validation dataset division (7:3) and features Z-score normalization were performed. Z-score normalization was used to convert different data to the same order of magnitude, so as to reduce the impact of different protocols and different operators on the US images, and to ensure that radiomics features were comparable (32–34). The calculation formula of Z-score normalization was as follows (33): y = (x − µ)/σ. where µ is the mean and σ is the standard deviation.

After data preprocessing, two-sample t-test or Mann-Whitney U test was used to screen radiomic features with statistically significant differences (P < 0.05) in order to eliminate irrelevant features in the training dataset. Then, the Spearman rank sum test was used for correlation analysis to eliminate the redundancy. Among the features with correlation coefficient > 0.9, only one was retained and the rest was excluded. Finally, the least absolute shrinkage and selection operator (LASSO) was performed, with penalty parameter tuning conducted by 5-fold cross-validation, to obtain the optimal regularization coefficient (lambda, also namely λ). Selected features with non-zero coefficients were analyzed with a linear regression model and weighted by their respective coefficients to construct a radiomics signature, called Radscore.

#### 2.6.2 Nomogram construction and model validation

In this study, 192 patients were randomly split into a training dataset (n = 134) and a validation dataset (n = 58). Univariate analyses were performed for the baseline patient characteristics, including clinic_CUS features and Radscore. Chi-square test or Fisher’s exact test was applied for categorical variables for training and validation datasets, while two-sample t-test or Mann-Whitney U test was used for continuous variables.

Univariable logistic regression analysis was performed to evaluate clinical information and CUS features (called clinic_CUS features), and to determine the potential predictor for LVI status in training dataset. And variables with P < 0.05 were subjected to subsequent multivariable logistic regression analysis, with a backward stepwise elimination method using the Akaike information criterion (AIC). The clinic_CUS features with P < 0.05 were confirmed as the final independent predictors to construct the clinic_CUS prediction model for LVI status. Then the Radscore and independent clinic_CUS features were combined, through multivariable logistic regression, to establish a radiomics nomogram prediction model. The variance inflation factor (VIF) was applied to estimate the collinearity diagnosis.

The potential predictive value of the established model was performed by receiver operating characteristic (ROC) curve analysis. The DeLong test was applied to compare differences between the areas under the curve (AUC). The goodness-of-fit of the radiomics nomogram was evaluated by Hosmer-Lemeshow test. We bootstrapped the data 1000 times to perform internal verification, drawing a calibration curve to verify the consistency between actual probability and the predicted probability. Decision curve analysis (DCA) was developed to ascertain the clinical utility of the radiomics nomogram by quantifying the net benefits at different threshold probabilities in the training and validation datasets (18). All procedures of building and validating radiomics nomogram model were shown in **Figure 1B**.

### 2.7 Statistical analysis

All statistical analyses were performed with R software (version4.1.3, http://www.Rproject.org) and SPSS software (version 26.0; Chicago, IL). Quantitative variables were expressed as mean ± standard deviation or medians (25% quantile, 75% quantile). Kolmogorov-Smirnov test and bartlett.test were used to evaluate the normality and homovariance of quantitative variables, so as to determine whether to use independent samples t-test or Mann Whitney U-test. Categorical data was analysed by chi-square test, or Fisher’s test. Intraclass correlation coefficient (ICC) was calculated to evaluate the agreement between intra- and inter-readers in the ROI delineation for radiomics feature extraction. Univariate and multivariate logistic regression were respectively used to select the significant predictors for building prediction models. The diagnostic performance to differentiate LVI status was performed by the area under the receiver operating curve (AUC) and the corresponding sensitivity, specificity, positive predictive value (PPV), negative predictive value (NPV), and accuracy. According to the AUC value, the diagnostic performance can be considered as high (AUC > 0.9), moderate (AUC = 0.7 ~ 0.9), or low (AUC = 0.5 ~ 0.7). Delong tests were performed to assess the differences in AUC values between different radiomics models. Hosmer-Lemeshow test was used to evaluate the goodness-of-fit of the radiomics nomogram. The calibration curve was applied to evaluate the consistency between actual probability and the predicted probability. Decision curve analysis (DCA) was developed to ascertain the clinical utility of different models. A 2-sided P value < 0.05 was considered a statistically significant difference. All packages of R4.1.3 that were listed in **Table 2**.

**Table 2:** Major packages of R software applied in current study.

Procedure of statistical analysis	R package
LASSO regression	glmnet
Univariate logistic regression	glm
Multivariate logistic regression	glm
Nomogram Construction	rms
Drawing ROC curves and measuring the AUC values	pROC
Calibration curves	rms
Decision curve analysis (DCA)	rmda

## 3 Results

### 3.1 Comparison of clinic_CUS features and construction of clinic_CUS model

Among the 192 patients with pT1 IDC, there were LVI-positive (n = 56) and LVI-negative (n = 136) cases. Included patients were divided into the training dataset (37 LVI -positive and 97 LVI -negative pT1 IDCs) and validation dataset (19 LVI -positive and 39 LVI -negative pT1 IDCs) randomly.

There were no significant differences in all the clinic_CUS features between the training dataset and the validation dataset (P = 0.131 ~ 0.940) (**Table 3**). The clinic_CUS features between LVI-positive and LVI-negative groups in the training and validation datasets are shown in **Table 4**.

**Table 3:** Comparisons of baseline characteristics in the training and validation datasets.

Characteristics	Training dataset (n = 134)	Validation dataset (n = 58)	P-value
LVI status, n (%)			0.471
LVI-negative	97 (72.4)	39 (67.2)	
LVI-positive	37 (27.6)	19 (32.8)	
Age, years	54.00 (47.00, 62.00)	55.03 ± 10.69	0.554
BMI, n (%)			0.935
≤24kg/m^2^	84 (62.7)	36 (62.1)	
>24kg/m^2^	50 (37.3)	22 (37.9)	
CA125, n (%)			0.940
0-35U/mL	122 (91)	53 (91.4)	
>35U/mL	12 (9)	5 (8.6)	
CA153, n (%)			0.850
<25U/mL	122 (91)	54 (93.1)	
≥25U/mL	12 (9)	4 (6.9)	
CEA, n (%)			0.632
<4μg/L	123 (91.8)	52 (89.7)	
≥4μg/L	11 (8.2)	6 (10.3)	
Tumor location, n (%)			0.211
Upper outer quadrant	58 (43.3)	20 (34.5)	
Lower outer quadrant	19 (14.2)	9 (15.5)	
Upper inner quadrant	28 (20.9)	21 (36.2)	
Lower inner quadrant	14 (10.4)	4 (6.9)	
Others	15 (11.2)	4 (6.9)	
Tumor size	1.64 ± 0.43	1.73 ± 0.34	0.324
Shape, n (%)			0.629
Regular	32 (23.9)	12 (20.7)	
Irregular	102 (76.1)	46 (79.3)	
Margin, n (%)			0.131
Circumscribed	28 (20.9)	18 (31)	
Not circumscribed	106 (79.1)	40 (69)	
Orientation, n (%)			0.574
Parallel	113 (84.3)	47 (81)	
Not parallel	21 (15.7)	11 (19)	
Echo pattern, n (%)			0.175
Hypoechoic	45 (33.6)	13 (22.4)	
Isoechoic or hyperechoic	4 (3)	4 (6.9)	
Heterogeneous	85 (63.4)	41 (70.7)	
Microcalcifications, n (%)			0.617
Absent	56 (41.8)	22 (37.9)	
Present	78 (58.2)	36 (62.1)	
Posterior features, n (%)			0.862
None	27 (20.1)	10 (17.2)	
Enhancement	34 (25.4)	18 (31)	
Shadowing	50 (37.3)	20 (34.5)	
Combined pattern	23 (17.2)	10 (17.2)	
US_LNM_status, n (%)			0.468
Absent	110 (82.1)	45 (77.6)	
Present	24 (17.9)	13 (22.4)	
Histological grade, n (%)			0.921
Grade 1	10 (7.5)	4 (6.9)	
Grade 2	84 (62.7)	35 (60.3)	
Grade 3	40 (29.9)	19 (32.8)	
ER status, n (%)			0.510
Negative	42 (31.3)	21 (36.2)	
Positive	92 (68.7)	37 (63.8)	
PR status, n (%)			0.462
Negative	57 (42.5)	28 (48.3)	
Positive	77 (57.5)	30 (51.7)	
HER-2 status, n (%)			0.244
Negative	101 (75.4)	39 (67.2)	
Positive	33 (24.6)	19 (32.8)	
Ki-67 status, n (%)			0.261
Low proliferation	23 (17.2)	14 (24.1)	
High proliferation	111 (82.8)	44 (75.9)	
Molecular subtype, n (%)			0.412
Luminal A	19 (14.2)	12 (20.7)	
Luminal B	75 (56)	26 (44.8)	
HER-2-positive	16 (11.9)	10 (17.2)	
Triple-negative	24 (17.9)	10 (17.2)	
Radscore	-1.01 ± 0.49	-0.93 (-1.52, -0.54)	0.806

**Table 4:** Comparisons of baseline characteristics between LVI-positive and LVI-negative groups in the training and validation datasets.

Characteristics	Training dataset (n=134)			Validation dataset (n=58)		
	LVI-negative (n = 97)	LVI-positive (n = 37)	P-value	LVI-negative (n = 39)	LVI-positive (n = 19)	P-value
Age, years	54.81 ± 12.12	52.22 ± 9.76	0.245	57.23 ± 10.36	50.53 ± 10.19	0.024
BMI, n (%)			0.013			0.107
≤24kg/m^2^	67 (69.1)	17 (45.9)		27 (69.2)	9 (47.4)	
>24kg/m^2^	30 (30.9)	20 (54.1)		12 (30.8)	10 (52.6)	
CA125, n (%)			0.271			1
0-35U/mL	90 (92.8)	32 (86.5)		36 (92.3)	17 (89.5)	
>35U/mL	7 (7.2)	5 (13.5)		3 (7.7)	2 (10.5)	
CA153, n (%)			0.031			0.189
<25U/mL	92 (94.8)	30 (81.1)		38 (97.4)	16 (84.2)	
≥25U/mL	5 (5.2)	7 (18.9)		1 (2.6)	3 (15.8)	
CEA, n (%)			0.083			0.623
<4μg/L	92 (94.8)	31 (83.8)		36 (92.3)	16 (84.2)	
≥4μg/L	5 (5.2)	6 (16.2)		3 (7.7)	3 (15.8)	
Tumor location, n (%)			0.661			0.966
Upper outer quadrant	44 (45.4)	14 (37.8)		14 (35.9)	6 (31.6)	
Lower outer quadrant	14 (14.4)	5 (13.5)		6 (15.4)	3 (15.8)	
Upper inner quadrant	21 (21.6)	7 (18.9)		14 (35.9)	7 (36.8)	
Lower inner quadrant	8 (8.2)	6 (16.2)		3 (7.7)	1 (5.3)	
Others	10 (10.3)	5 (13.5)		2 (5.1)	2 (10.5)	
Tumor size, cm	1.62 ± 0.44	1.69 ± 0.41	0.416	1.69 ± 0.36	1.82 ± 0.29	0.172
Shape, n (%)			0.941			1
Regular	23 (23.7)	9 (24.3)		8 (20.5)	4 (21.1)	
Irregular	74 (76.3)	28 (75.7)		31 (79.5)	15 (78.9)	
Margin, n (%)			0.025			0.018
Circumscribed	25 (25.8)	3 (8.1)		16 (41)	2 (10.5)	
Not circumscribed	72 (74.2)	34 (91.9)		23 (59)	17 (89.5)	
Orientation, n (%)			0.242			0.522
Parallel	84 (86.6)	29 (78.4)		33 (84.6)	14 (73.7)	
Not parallel	13 (13.4)	8 (21.6)		6 (15.4)	5 (26.3)	
Echo pattern, n (%)			0.706			0.577
Hypoechoic	35 (36.1)	10 (27)		10 (25.6)	3 (15.8)	
Isoechoic or hyperechoic	3 (3.1)	1 (2.7)		2 (5.1)	2 (10.5)	
Heterogeneous	59 (60.8)	26 (70.3)		27 (69.2)	14 (73.7)	
Microcalcifications, n (%)			0.567			0.203
Absent	42 (43.3)	14 (37.8)		17 (43.6)	5 (26.3)	
Present	55 (56.7)	23 (62.2)		22 (56.4)	14 (73.7)	
Posterior features, n (%)			0.512			0.731
None	21 (21.6)	6 (16.2)		6 (15.4)	4 (21.1)	
Enhancement	27 (27.8)	7 (18.9)		11 (28.2)	7 (36.8)	
Shadowing	34 (35.1)	16 (43.2)		14 (35.9)	6 (31.6)	
Combined pattern	15 (15.5)	8 (21.6)		8 (20.5)	2 (10.5)	
US_LNM_status, n (%)			< 0.001			0.030
Absent	90 (92.8)	20 (54.1)		34 (87.2)	11 (57.9)	
Present	7 (7.2)	17 (45.9)		5 (12.8)	8 (42.1)	
Histological grade, n (%)			0.816			0.167
Grade 1	8 (8.2)	2 (5.4)		4 (10.3)	0 (0)	
Grade 2	61 (62.9)	23 (62.2)		25 (64.1)	10 (52.6)	
Grade 3	28 (28.9)	12 (32.4)		10 (25.6)	9 (47.4)	
ER status, n (%)			0.559			0.944
Negative	29 (29.9)	13 (35.1)		14 (35.9)	7 (36.8)	
Positive	68 (70.1)	24 (64.9)		25 (64.1)	12 (63.2)	
PR status, n (%)			0.919			0.512
Negative	41 (42.3)	16 (43.2)		20 (51.3)	8 (42.1)	
Positive	56 (57.7)	21 (56.8)		19 (48.7)	11 (57.9)	
HER-2 status, n (%)			0.397			0.290
Negative	75 (77.3)	26 (70.3)		28 (71.8)	11 (57.9)	
Positive	22 (22.7)	11 (29.7)		11 (28.2)	8 (42.1)	
Ki-67 status, n (%)			0.739			0.173
Low proliferation	16 (16.5)	7 (18.9)		12 (30.8)	2 (10.5)	
High proliferation	81 (83.5)	30 (81.1)		27 (69.2)	17 (89.5)	
Molecular subtype, n (%)			0.694			0.341
Luminal A	13 (13.4)	6 (16.2)		10 (25.6)	2 (10.5)	
Luminal B	57 (58.8)	18 (48.6)		15 (38.5)	11 (57.9)	
HER-2-positive	10 (10.3)	6 (16.2)		6 (15.4)	4 (21.1)	
Triple-negative	17 (17.5)	7 (18.9)		8 (20.5)	2 (10.5)	
Radscore	-1.14 ± 0.46	-0.67 ± 0.41	< 0.001	-1.26 (-1.63, -0.83)	-0.68 ± 0.38	0.001

In the univariable logistic regression analysis for clinic_CUS features, the initially selected risk predictors included BMI, CA153, CEA, tumor margin, US_LNM_status (all P < 0.05) (**Table 5**).

**Table 5:** Univariate logistic analysis for the risk factors of LVI.

Characteristics	OR	95%CI	P-value
age	0.98	0.948-1.014	0.245
BMI			
≤24kg/m^2^	Reference		
>24kg/m^2^	2.627	1.209-5.71	0.015
CA125			
0-35U/mL	Reference		
>35U/mL	2.009	0.595-6.785	0.261
CA153			
<25U/mL	Reference		
≥25U/mL	4.293	1.269-14.53	0.019
CEA			
<4μg/L	Reference		
≥4μg/L	3.561	1.016-12.485	0.047
Tumor_location			
Upper outer quadrant	Reference		
Lower outer quadrant	1.122	0.343-3.674	0.848
Upper inner quadrant	1.048	0.368-2.984	0.931
Lower inner quadrant	2.357	0.698-7.961	0.167
Others	1.571	0.459-5.381	0.472
Tumor size	1.457	0.59-3.597	0.414
Shape			
Regular	Reference		
Irregular	0.967	0.399-2.341	0.941
Margin			
Circumscribed	Reference		
Not circumscribed	3.935	1.112-13.932	0.034
Orientation			
Parallel	Reference		
Not parallel	1.782	0.672-4.731	0.246
Echo pattern			
Hypoechoic	Reference		
Isoechoic or hyperechoic	1.167	0.109-12.476	0.899
Heterogeneous	1.542	0.665-3.576	0.312
Microcalcifications			
Absent	Reference		
Present	1.255	0.577-2.726	0.567
Posterior features			
None	Reference		
Enhancement	0.907	0.265-3.107	0.877
Shadowing	1.647	0.557-4.869	0.367
Combined pattern	1.867	0.536-6.506	0.327
US_LNM_status			
Absent	Reference		
Present	10.929	3.998-29.87	<0.001
Histological grade			
Grade 1	Reference		
Grade 2	1.508	0.298-7.643	0.62
Grade 3	1.714	0.316-9.304	0.532
ER status			
Negative	Reference		
Positive	0.787	0.352-1.759	0.559
PR status			
Negative	Reference		
Positive	0.961	0.447-2.064	0.919
HER-2 status			
Negative	Reference		
Positive	1.442	0.616-3.377	0.398
Ki-67 status			
Low proliferation	Reference		
High proliferation	0.847	0.317-2.26	0.74
Molecular_subtype			
Luminal A	Reference		
Luminal B	0.684	0.227-2.063	0.5
HER-2-positive	1.3	0.321-5.269	0.713
Triple-negative	0.892	0.241-3.298	0.864
Radscore	11.808	3.924-35.526	<0.001

OR, Odds ratio; 95% CI, 95% confidence interval

After the multivariable logistic regression analysis, tumor margin and US_LNM_status were regarded as independent clinic_CUS risk predictors for the LVI status (all P < 0.05) (**Table 6**). The VIF value of the two predictors was 1.177, indicating no significant collinearity.

**Table 6:** Multivariate logistic analysis for the risk factors of LVI.

Characteristics	Clinic_CUS model			Radiomics nomogram model		
	OR	95%CI	P-value	OR	95%CI	P-value
Margin						
Circumscribed	Reference			Reference		
Not circumscribed	7.893	1.658-37.566	0.009	6.524	1.272-33.45	0.025
US_LNM_status						
Absent	Reference			Reference		
Present	15.034	4.53-49.889	<0.001	12.92	3.537-47.196	<0.001
Radscore	NA	NA	NA	7.778	2.486-24.336	<0.001

OR, Odds ratio; 95% CI, 95% confidence interval. NA, not available.

### 3.2 Feature selection and Radscore establishment

In this study, a total of 939 radiomics features for each patient were extracted from grayscale US images. We selected 858 features with high stability and reproducibility (ICCs > 0.75) for subsequent feature screening as follows.

Step 1: After the two-sample t-test or Mann-Whitney U test, 103 features remained.

Step 2: After eliminating redundancy by applying Spearman correlation analysis, the number of remaining features was 35.

**Figure 2:**
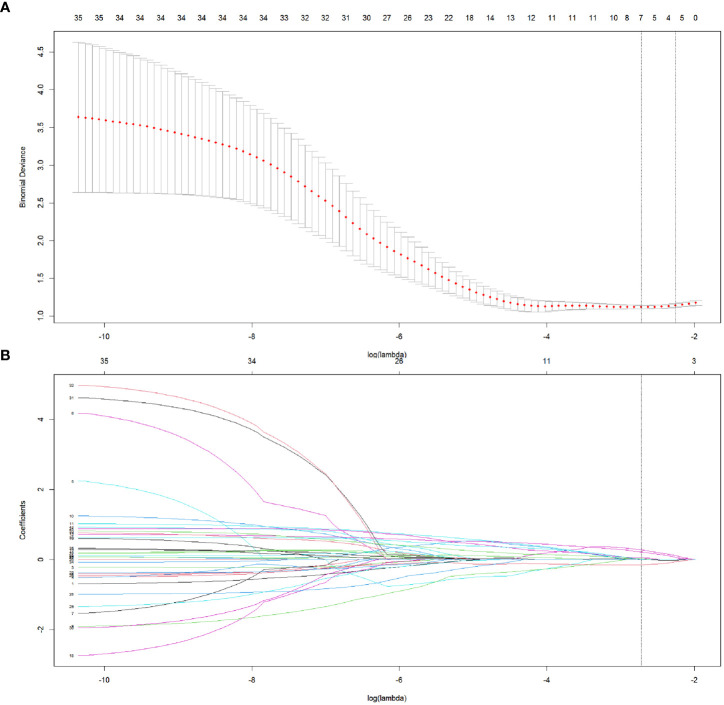
The selection of radiomics features in LASSO regression. **(A)** The association between binomial deviance and log (lambda) was plotted, and two vertical line were drawn *via* the minimum deviance and 1 standard error of the minimum deviance, respectively. Based on the minimum criteria, the optimal lambda was selected according to 5-fold cross-validation. **(B)** LASSO coefficient distribution of radiomics features by different log (lambda) values. The vertical line was drawn at the optimal value of λ, leading to 7 features with nonzero coefficients.

**Figure 3:**
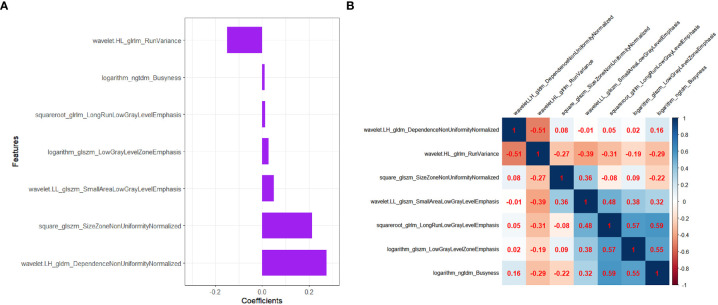
The selected radiomics features to construct the Radscore. **(A)** The coefficients of radiomics features in the construction of Radscore. **(B)** Heatmap depicting correlation coefficient matrix of the selected radiomics features associated with LVI. The degree of correlation between various features is shown in different shades of color.

Step 3: The features were reduced to 7 potential predictors with nonzero coefficients in the LASSO regression model, and the optimal lambda (λ) was chosen as 0.066 (**Figure 2A, B**; **Figure 3A**). The correlation analysis showed that the maximum correlation coefficient between the selected radiomic features was 0.59 (**Figure 3B**).

The final remaining 7 radiomics features were incorporated into the calculation of Radscore with the following formula:


$$\begin{array}{l} \text{Radscore}=(-1.014)+0.275*\text{wavelet.LH\_gldm\_DependenceNonUniformityNormalized}+\;\left(-0.151\right)*\text{wavelet.HL\_glrlm\_RunVariance}+\\ 0.213*\text{square\_glszm\_SizeZoneNonUniformityNormalized}+0.049*\text{wavelet.LL\_glszm\_SmallAreaLowGrayLevelEmphasis}+\\ 0.013*\text{squareroot\_glrlm\_LongRunLowGrayLevelEmphasis}+\;0.028*\text{logarithm\_glszm\_LowGrayLevelZoneEmphasis}+0.010*\text{logarithm\_ngtdm\_Busyness} \end{array}$$

**Figure 4:**
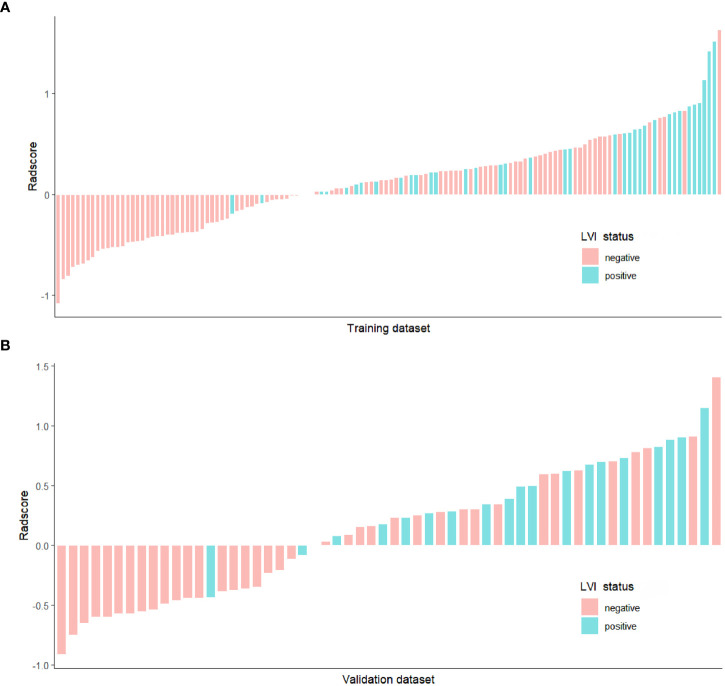
Bar chart of Radscore in the training **(A)** and validation **(B)** datasets, and the y-axis refers to the Radscore minus the cut-off value (i.e., Radscore +1.138). Up and down bars refer to the predicted positive LVI and negative LVI status, respectively. Green and red bars represent the actual LVI status as positive and negative, respectively.

There was no significant difference for Radscore between the training dataset and the validation dataset (P = 0.806)(**Table 3**). In both training and validation datasets, the LVI-positive group had significantly higher Radscores than those of the LVI-negative group (P < 0.001 and P=0.001, repectively). (**Table 4**). The optimal cut-off value of the Radscore for discriminating LVI status was -1.138 in the training dataset. We used this cut-off value to plot Radscore bar chart in the training (**Figure 4A**) and validation (**Figure 4B**) datasets.

The Radscore presented good discrimination performance in predicting the status of LVI in both training and validation datasets (**Table 7**).

**Table 7:** Diagnostic performance of the Radscore, clinical_CUS, and radiomics nomogram models in the training and validation datasets.

Prediction models	Datasets	Cut-off value	AUC	95% CI		ACC	SEN	SPE	PPV	NPV
				Lower	Upper					
Radscore	Training	-1.138	0.775	0.693	0.857	0.627	0.946	0.505	0.422	0.961
	Validation	-1.138	0.768	0.647	0.889	0.655	0.895	0.539	0.486	0.913
Clinic_CUS	Training	0.295	0.747	0.666	0.828	0.799	0.459	0.928	0.708	0.818
	Validation	0.295	0.713	0.579	0.848	0.724	0.421	0.872	0.615	0.756
Radiomics nomogram	Training	0.390	0.849	0.775	0.923	0.858	0.649	0.938	0.800	0.875
	Validation	0.390	0.854	0.748	0.960	0.810	0.684	0.872	0.722	0.850

95% CI, 95% confidence interval; ACC, accuracy; SEN, sensitivity; SPE, specificity; PPV, positive predictive value; NPV, negative predictive value.

### 3.3 Construction and validation of radiomics nomogram

**Figure 5:**
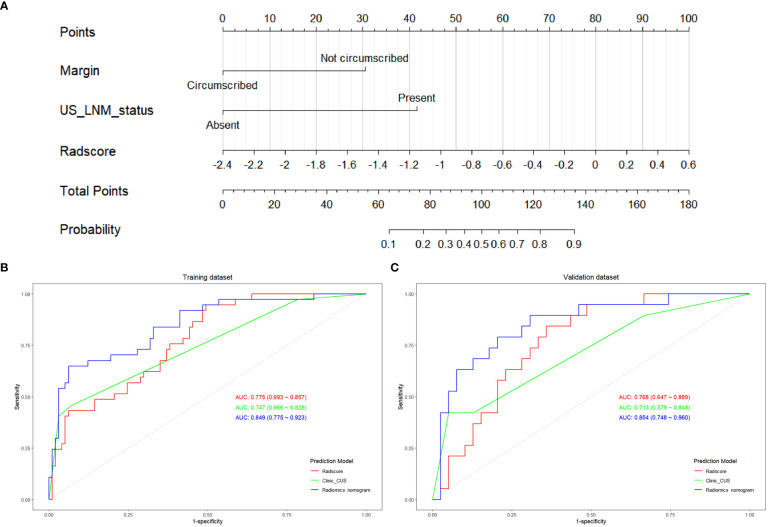
The construction of radiomics nomogram and the ROC curves of Radscore, clinic_CUS, and radiomics nomogram models. **(A)** Radiomics nomogram model visualized and constructed with tumor margin, US_LNM status and Radscore. The ROC curves of the Radscore model, clinic_CUS model, and radiomics nomogram model in training **(B)** and validation **(C)** datasets.

On multivariate logistic regression analysis for clinic_CUS features and Radscore, tumor margin, US_LNM_status and Radscore were identified as independent risk factors for predicting LVI status (**Table 6**). The VIF values of the three predictors were 1.000 ~ 1.175, indicating no significant collinearity. Therefore, tumor margin, US_LNM_status and Radscore constructed the preoperative radiomics nomogram model (**Figure 5A**).

The radiomics nomogram score (Nomoscore) for each patient was calculated using the following formula: Nomoscore = -1.228 + 1.875* margin+2.559×US_LNM_status+ 2.051× Radscore

The ROC curve indicated that the radiomics nomogram model had satisfactory performance and applicability with the AUC value of 0.849 (95% CI: 0.775 ~ 0.923) in training dataset and 0.854 (95% CI: 0.748 ~ 0.960) in validation dataset (**Table 7**). DeLong test was used to compare AUC values between Radscore, clinic_CUS model, and the radiomics nomogram model. In the training dataset, the radiomics nomogram model was significantly superior to the clinical model and Radscore model (P < 0.001 and P = 0.014, respectively). In the validation dataset, the radiomics nomogram model was significantly superior to the clinical model (P = 0.001), but not significantly superior to Radscore (P = 0.165). No significant difference in AUC values was found between the Radscore and the clinic_CUS model in the training dataset (P = 0.598) and in validation dataset (P = 0.567). The ROC curves of the three models in the training and validation datasets are shown in **Figures 5B, C**, respectively.

**Figure 6:**
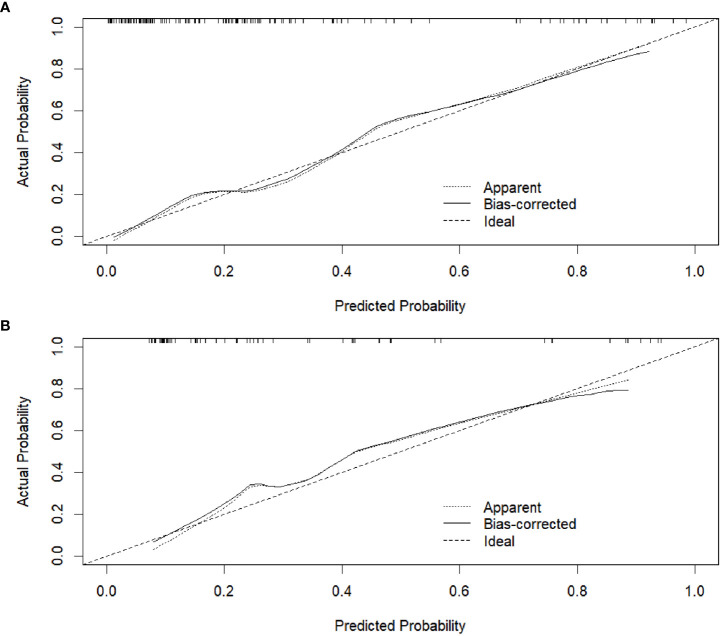
Calibration curves of the radiomics nomogram in the training dataset **(A)** and validation dataset **(B)**, respectively.

The calibration curve of the radiomics nomogram showed good agreement between the predicted LVI probabilities and the actual LVI status in both training and validation datasets (**Figures 6A, B**). The results of the Hosmer-Lemeshow test were not significant in both training and validation datasets (P = 0.550 and 0.812, respectively), indicating favorable goodness-of-fit of the radiomics nomogram.

**Figure 7:**
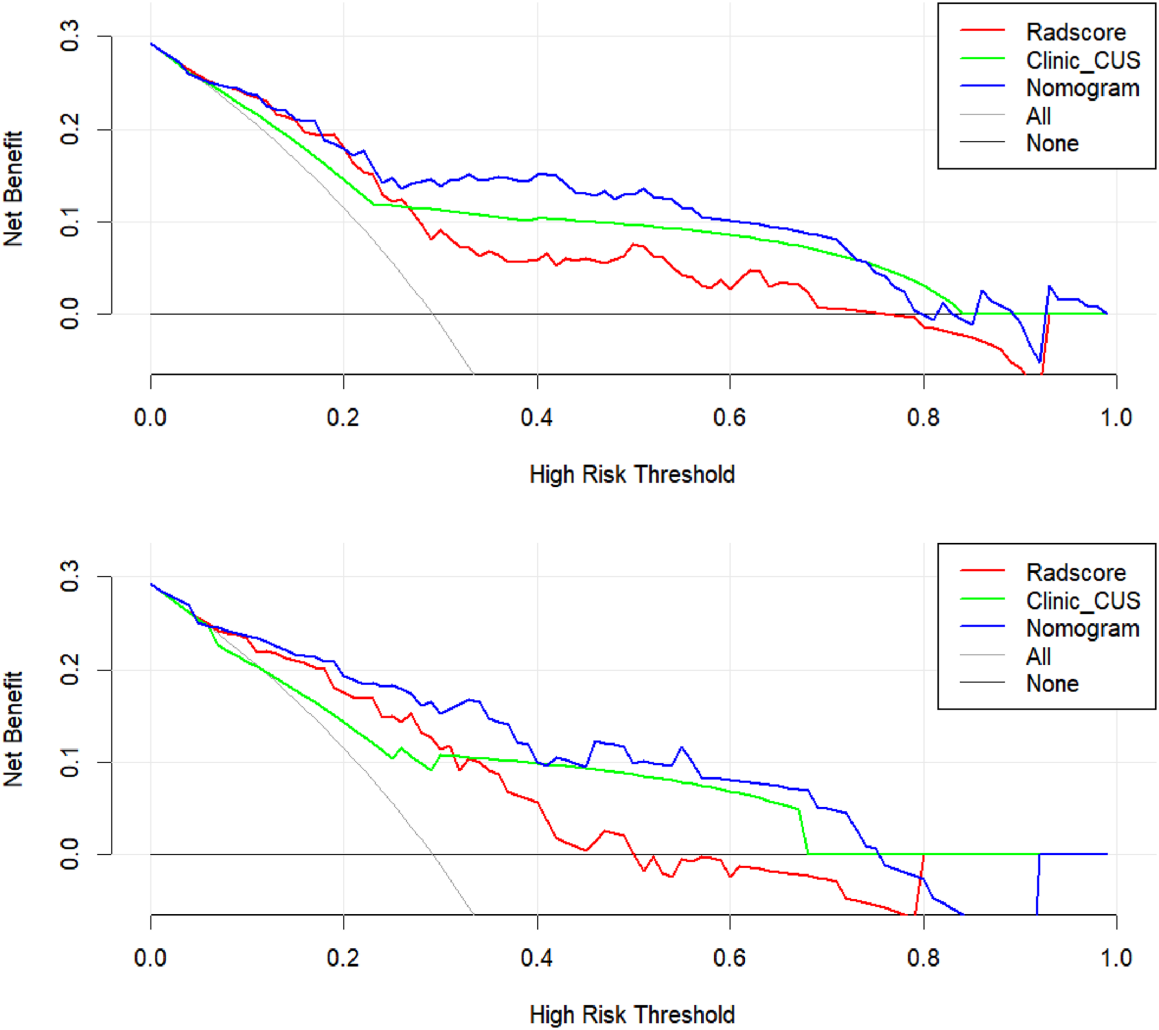
DCA for the clinical value assessment of the three models in the training dataset **(A)** and validation dataset **(B)**, respectively. The X-axis indicates the threshold probability, and the Y-axis represents the net benefit. The gray line indicates the assumption that all patients are LVI-positive cases; the black line represents the assumption that all patients are LVI-negative cases. The red, green, and blue lines refer to the Radscore, clinic_CUS, and radiomics nomogram models, respectively.

The DC showed that the radiomics nomogram model had higher net benefit than the Radscore and clinic_CUS models in predicting LVI in the training dataset (**Figure 7A**), when threshold probabilities ranged from 0.21 to 0.73. Similar results presented in the validation dataset (**Figure 7B**). The DCA demonstrated the clinical value of the radiomics nomogram model.

## 4 Discussion

In our study, a Radscore derived from grayscale US image was developed and validated to predict the LVI status in patients with pT1 IDC. Moreover, the radiomics nomogram combining tumor margin, US_LNM and Radscore provides a direct, non-invasive way for preoperative prediction of LVI while exhibiting high accuracy in the individualized prediction of LVI.

T1 stage breast cancer has become the most commonly diagnosed invasive breast disease in developed countries (23). The incidence of LVI in T1 breast cancer is 13% ~ 27% (5). Among the 192 patients with pathologically confirmed T1 breast cancer in our study, the incidence of LVI is 29.2% (56/192), which is slightly higher than the results of previous studies. This may be related to the fact that all breast cancers included in this study were invasive ductal carcinoma. LVI is defined as the invasion of lymphatic vessel walls by tumor cells or the spread of carcinoma thrombus into the luminal cavity, which had been considered as a critical step in the relapse or progression of disseminated tumor cells (1, 6, 7). LVI was highlighted as a risk factor with important implications for survival in patients with T1 breast cancer (9, 10). The presence of LVI is not always able to be detected at biopsy, owing to the small tissue volume to be cut and obtained from the primary tumor (3, 12, 13); therefore, noninvasive prediction of LVI is necessary.

Previously, it had been demonstrated that the clinic_CUS features of breast carcinoma could distinguish LVI-positive from LVI-negative (8, 12). In our study, tumor margin and US_LNM status were independent predictors of LVI in the clinic_CUS model. Tumor margin showed significant difference in LVI-positive group and LVI-negative group, and LVI-positive breast tumors were more likely to present with not circumscribed margins than LVI-negative tumors. This is consistent with previous studies (6, 12, 22). Reasonable explanations may be that tumor microvascular invasion usually occurred in the peritumoral area, while undefined margin, especially spiculate margin, was associated with the infiltrative growth of the tumor into the peritumoral stroma (6). As another independent predictor for LVI in the clinic_CUS model, US_LNM was more common in LVI-positive than LVI-negative breast cancers. Previous studies (6–9) had also demonstrated that the presence of LVI was closely associated with axillary lymph node involvement, which were consistent with our study. Similarly, ZHOU et al. (12) and Tong et al. (8) all thought that suspicious findings on axillary US imaging were independent predictor for the presence of LVI. Moreover, LVI-positive were more likely to have nodal metastases than LVI-negative in T1 breast cancer patients (9), whereas lymph node metastases were naturally relatively low in T1 breast cancer (35), perhaps more likely to be LVI-positive at the time of lymph node metases.

Recently, some studies proposed that MRI-based and digital breast tomosynthesis (DBT)-based radiomis score were independent risk factors for predicting LVI status of breast cancer patients (3, 6, 7, 11). Their results demonstrated good predictive efficacy for LVI status of breast cancer. Compared with the prior MRI or DBT, grayscale US images were simpler and easier to obtain for radiomics analysis. In addition, US-based radiomics score had been shown to be of great value for the discrimination and prognostic assessment of breast tumor (18–21), but had rarely been used to predict LVI, especially for small breast cancer. Therefore, we chose to use the US image for radiomics analysis in order to discover its potential value for predicting LVI in T1 stage IDC. In our study, Radscore was calculated by 7 radiomics features associated with the LVI status in the construction of Radscore model. All the selected 7 radiomics features were from transform-filtered texture features, including 2 GLRLM features, 1 NGTDM feature, 3 GLSZM features, and 1 GLDM feature, from which wavelet.LH_gldm_DependenceNonUniformityNormalized and square_glszm_SizeZoneNonUniformityNormalized were the dominant features in the construction of the Radscore.

The texture features could quantify the spatial variation in the architecture and function of breast cancer, which are suitable to assess the information of tumor heterogeneity (20, 34). The transform-filtered texture features could provide potential insight for quantifying tumor biological and multidimensional heterogeneity (6, 11, 30, 36). Many studies had found that transform-filtered texture features were useful in predicting the tumor benignity and malignancy, lymph node metastasis, gene expression, and the efficacy of neoadjuvant chemotherapy (11, 18–20, 30, 36, 37), although some explanations of relationship between these complex features and tumor biology behavior remained to be elucidated (6). Some transform-filtered texture features from DBT and MRI could be incorporated as prediction model for the LVI status in breast cancer (6, 11), since the transform-filtered features may be associated with the tumor complex microstructure, such as tumor cell proliferation, local necrosis, hemorrhage, inflammation, and microcalcifications, etc in LVI-positive tumor (6, 7). Different imaging modalities contained different potential information about the microstructure and biological behavior of the tumor (6), thus US-based Radscore could also had certain value in predicting LVI in breast cancer. Our results revealed that LVI-positive pT1 IDCs exhibited higher scores on the grayscale US image based Radscore model, which may be attributed to the higher invasiveness and heterogeneity of LVI-positive tumors (6, 11).

US-based radiomics nomogram had been widely performed and demonstrated to be of great clinical value in oncology (18, 20, 21, 36, 38, 39). To our knowledge, our study is the first to utilize the US-based radiomics nomogram to preoperatively predict the LVI status in pT1 IDC. In this study, we developed and validated a US-based radiomics nomogram, constructed with three independent predictors, namely tumor margin, US_LNM status and Radscore. Our result revealed that the radiomics nomogram model, incorporating tumor margin, US_LNM status and US-based Radscore, exhibited a good discrimination ability for predicting LVI in pT1 IDC, with the AUC value of 0.849 (95% CI: 0.775~0.923) in the training dataset, and 0.854 (95% CI: 0.748~0.960) in the validation dataset. In addition, the radiomics nomogram model presented superior predictive performance to the clinical model both in training and validation datasets, and the radiomics nomogram model could be more intuitive and easily applied in distinguishing LVI status in pT1 IDC. The predictive performance of LVI in our research was similar to that in previous studies (6, 7, 11), which were based on MRI or DBT for radiomics nomogram construction. Moreover, the calibration curve for our radiomics nomogram model showed a good concordance between predicted and actual probability, and DCA demonstrated a higher clinical net benefit from the nomogram model. Thus, we strongly believe that the constuction of radiomics nomogram based on US image can be effectively applied to predict the status of LVI in pT1 IDC. Future research directions would be associated with other prognostic indicators or treatment assessment of breast carcinoma through US-based radiomics nomogram.

Our study has several limitations. Firstly, this study only included pT1 IDC. Other histological types or T stages of breast cancer would be included to increase the clinical applicability of this study. Secondly, this is a single center retrospective study, in which case selection bias may exist. Moreover, the sample size of this study is not large enough, so a larger sample and multicenter data would be deserved in the future. Thirdly, we manually delineated the maximum diameter level of the tumor as two-dimensional (2D) ROI, which could not represent the whole tumor. Fourthly, we only based on the grayscale US images for radiomics analysis. Multimodal ultrasound images, including elastography and CEUS imaging, would be performed in the future study. Fifthly, US images were acquired from multiple manufacturers in this study. Although we standardize the images before feature extraction and performed Z-score normalization for radiomics features, heterogeneity of images from different operators and different equipments may also have certain impact on experimental results. Finally, LVI usually indicates tumor invasion in peritumoral tissue, but radiomic analysis of the peritumoral ROI was not performed. Further studies are needed to verify this aspect as well.

In conclusion, radiomics features derived from grayscale US image may be potential biomarkers for predicting LVI of pT1 IDC. The proposed radiomics nomogram, incorporating tumor margin, US_LNM status and US-based Radscore, could provide a satisfactory predictive efficacy for LVI status and confer a higher clinical benefit for patients.

## Data availability statement

The raw data supporting the conclusions of this article are available from the corresponding authors on reasonable request.

## Ethics statement

The studies involving human participants were reviewed and approved by Hubei Cancer Hospital (No.: LLHBCH2021YN-001). Written informed consent for participation was not required for this study in accordance with the national legislation and the institutional requirements. Written informed consent was not obtained from the individual(s) for the publication of any potentially identifiable images or data included in this article.

## Author contributions

FL, X-WC, and G-FL conceived and designed of the study. FL and S-EZ acquired the data. M-LX, FL, and S-EZ analyzed and/or interpreted of the data. M-LX and FL drafted the manuscript. M-LX, FL, X-WC, and G-FL revised the manuscript critically for important intellectual content. All authors contributed to the article and approved the submitted version.

## References

[1] Choi BB. Dynamic contrast enhanced-mri and diffusion-weighted image as predictors of lymphovascular invasion in node-negative invasive breast cancer. World J Surg Oncol (2021) 19:76. doi: 10.1186/s12957-021-02189-3 33722246 PMC7962354

[2] Ryu YJ Kang SJ Cho JS Yoon JH Park MH. Lymphovascular invasion can be better than pathologic complete response to predict prognosis in breast cancer treated with neoadjuvant chemotherapy. Med (Baltimore) (2018) 97:e11647. doi: 10.1097/MD.0000000000011647 PMC6078671 30045313

[3] Kayadibi Y Kocak B Ucar N Akan YN Yildirim E Bektas S. Mri radiomics of breast cancer: machine learning-based prediction of lymphovascular invasion status. Acad Radiol (2022) 29:S126–34. doi: 10.1016/j.acra.2021.10.026 34876340

[4] Nijiati M Aihaiti D Huojia A Abulizi A Mutailifu S Rouzi N. Mri-based radiomics for preoperative prediction of lymphovascular invasion in patients with invasive breast cancer. Front Oncol (2022) 12:876624. doi: 10.3389/fonc.2022.876624 35734595 PMC9207467

[5] Lyu Z Wang J Kang L Hu C He H Guan M. Lymph node metastasis and prognostic analysis of 354 cases of t1 breast cancer. Zhōnghuá zhongliú zázhì (2014) 36:382–5. doi: 10.3760/cma.j.issn.0253-3766.2014.05.013 25030596

[6] Wang D Liu M Zhuang Z Wu S Zhou P Chen X. Radiomics analysis on digital breast tomosynthesis: preoperative evaluation of lymphovascular invasion status in invasive breast cancer. Acad Radiol (2022) 29:17732–82. doi: 10.1016/j.acra.2022.03.011 35400556

[7] Liu Z Feng B Li C Chen Y Chen Q Li X. Preoperative prediction of lymphovascular invasion in invasive breast cancer with dynamic contrast-enhanced-mri-based radiomics. J Magn Reson Imaging (2019) 50:847–57. doi: 10.1002/jmri.26688 30773770

[8] Tong YY Sun PX Zhou J Shi ZT Chang C Li JW. The association between ultrasound features and biological properties of invasive breast carcinoma is modified by age, tumor size, and the preoperative axilla status. J Ultrasound Med (2020) 39:1125–34. doi: 10.1002/jum.15196 31875336

[9] Zhao Y Yang N Wang X Huang Y Zhou X Zhang D. Potential roles of lymphovascular space invasion based on tumor characteristics provide important prognostic information in t1 tumors with er and her2 positive breast cancer. Clin Transl Oncol (2020) 22:2275–85. doi: 10.1007/s12094-020-02369-9 32447641

[10] Houvenaeghel G Goncalves A Classe JM Garbay JR Giard S Charytensky H. Characteristics and clinical outcome of t1 breast cancer: a multicenter retrospective cohort study. Ann Oncol (2014) 25:623–8. doi: 10.1093/annonc/mdt532 PMC4433506 24399079

[11] Zhang J Wang G Ren J Yang Z Li D Cui Y. Multiparametric mri-based radiomics nomogram for preoperative prediction of lymphovascular invasion and clinical outcomes in patients with breast invasive ductal carcinoma. Eur Radiol (2022) 32:4079–89. doi: 10.1007/s00330-021-08504-6 35050415

[12] Zhou P Jin C Lu J Xu L Zhu X Lian Q. The value of nomograms in pre-operative prediction of lymphovascular invasion in primary breast cancer undergoing modified radical surgery: based on multiparametric ultrasound and clinicopathologic indicators. Ultrasound Med Biol (2021) 47:517–26. doi: 10.1016/j.ultrasmedbio.2020.11.007 33277109

[13] Igarashi T Furube H Ashida H Ojiri H. Breast mri for prediction of lymphovascular invasion in breast cancer patients with clinically negative axillary lymph nodes. Eur J Radiol (2018) 107:111–8. doi: 10.1016/j.ejrad.2018.08.024 30292254

[14] Cheung SM Husain E Mallikourti V Masannat Y Heys S He J. Intra-tumoural lipid composition and lymphovascular invasion in breast cancer *via* non-invasive magnetic resonance spectroscopy. Eur Radiol (2021) 31:3703–11. doi: 10.1007/s00330-020-07502-4 PMC8128855 33270144

[15] Liu Z Li R Liang K Chen J Chen X Li X. Value of digital mammography in predicting lymphovascular invasion of breast cancer. BMC Cancer (2020) 20:274. doi: 10.1186/s12885-020-6712-z 32245448 PMC7119272

[16] Xu M Li F Yu S Zeng S Weng G Teng P. Value of histogram of gray-scale ultrasound image in differential diagnosis of small triple negative breast invasive ductal carcinoma and fibroadenoma. Cancer Manag Res (2022) 14:1515–24. doi: 10.2147/CMAR.S359986 PMC9038159 35478712

[17] Rigiroli F Hoye J Lerebours R Lafata KJ Li C Meyer M. Ct radiomic features of superior mesenteric artery involvement in pancreatic ductal adenocarcinoma: a pilot study. Radiology (2021) 301:610–22. doi: 10.1148/radiol.2021210699 PMC9899097 34491129

[18] Du Y Zha H Wang H Liu X Pan J Du L. Ultrasound-based radiomics nomogram for differentiation of triple-negative breast cancer from fibroadenoma. Br J Radiol (2022) 95:20210598. doi: 10.1259/bjr.20210598 35138938 PMC10993963

[19] Jiang M Li CL Luo XM Chuan ZR Lv WZ Li X. Ultrasound-based deep learning radiomics in the assessment of pathological complete response to neoadjuvant chemotherapy in locally advanced breast cancer. Eur J Cancer (2021) 147:95–105. doi: 10.1016/j.ejca.2021.01.028 33639324

[20] Zha HL Zong M Liu XP Pan JZ Wang H Gong HY. Preoperative ultrasound-based radiomics score can improve the accuracy of the memorial sloan kettering cancer center nomogram for predicting sentinel lymph node metastasis in breast cancer. Eur J Radiol (2021) 135:109512. doi: 10.1016/j.ejrad.2020.109512 33429302

[21] Gao Y Luo Y Zhao C Xiao M Ma L Li W. Nomogram based on radiomics analysis of primary breast cancer ultrasound images: prediction of axillary lymph node tumor burden in patients. Eur Radiol (2021) 31:928–37. doi: 10.1007/s00330-020-07181-1 32845388

[22] Ouyang FS Guo BL Huang XY Ouyang LZ Zhou CR Zhang R. A nomogram for individual prediction of vascular invasion in primary breast cancer. Eur J Radiol (2019) 110:30–8. doi: 10.1016/j.ejrad.2018.11.013 30599870

[23] Zhao Y Liu Y Xie S Jiang Y Shao Z. A nomogram predicting lymph node metastasis in t1 breast cancer based on the surveillance, epidemiology, and end results program. J Cancer (2019) 10:2443–9. doi: 10.7150/jca.30386 PMC6584352 31258749

[24] Li Y Chen Y Zhao R Ji Y Li J Zhang Y. Development and validation of a nomogram based on pretreatment dynamic contrast-enhanced mri for the prediction of pathologic response after neoadjuvant chemotherapy for triple-negative breast cancer. Eur Radiol (2022) 32:1676–87. doi: 10.1007/s00330-021-08291-0 34767068

[25] Zheng X Yao Z Huang Y Yu Y Wang Y Liu Y. Deep learning radiomics can predict axillary lymph node status in early-stage breast cancer. Nat Commun (2020) 11:1236. doi: 10.1038/s41467-020-15027-z 32144248 PMC7060275

[26] Xu M Tang Q Li M Liu Y Li F. An analysis of ki-67 expression in stage 1 invasive ductal breast carcinoma using apparent diffusion coefficient histograms. Quant Imaging Med Surg (2021) 11:1518–31. doi: 10.21037/qims-20-615 PMC7930667 33816188

[27] Goldhirsch A Wood WC Coates AS Gelber RD Thurlimann B Senn HJ. Strategies for subtypes–dealing with the diversity of breast cancer: highlights of the st. gallen international expert consensus on the primary therapy of early breast cancer 2011. Ann Oncol (2011) 22:1736–47. doi: 10.1093/annonc/mdr304 PMC3144634 21709140

[28] Ji G Zhu F Xu Q Wang K Wu M Tang W. Radiomic features at contrast-enhanced ct predict recurrence in early stage hepatocellular carcinoma: A multi-institutional study. Radiology (2020) 294:568–79. doi: 10.1148/radiol.2020191470 31934830

[29] Sun R Limkin EJ Vakalopoulou M Dercle L Champiat S Han SR. A radiomics approach to assess tumour-infiltrating cd8 cells and response to anti-pd-1 or anti-pd-l1 immunotherapy: An imaging biomarker, retrospective multicohort study. Lancet Oncol (2018) 19:1180–91. doi: 10.1016/S1470-2045(18)30413-3 30120041

[30] Ren S Li Q Liu S Qi Q Duan S Mao B. Clinical value of machine learning-based ultrasomics in preoperative differentiation between hepatocellular carcinoma and intrahepatic cholangiocarcinoma: a multicenter study. Front Oncol (2021) 11:749137. doi: 10.3389/fonc.2021.749137 34804935 PMC8604281

[31] Zhang Y Luo Y Kong X Wan T Long Y Ma J. A preoperative mri-based radiomics-clinicopathological classifier to predict the recurrence of pituitary macroadenoma within 5 years. Front Neurol (2021) 12:780628. doi: 10.3389/fneur.2021.780628 35069413 PMC8767054

[32] Qin H Wu Y Lin P Gao R Li X Wang X. Ultrasound image-based radiomics: an innovative method to identify primary tumorous sources of liver metastases. J Ultrasound Med (2021) 40:1229–44. doi: 10.1002/jum.15506 32951217

[33] Peng Y Lin P Wu L Wan D Zhao Y Liang L. Ultrasound-based radiomics analysis for preoperatively predicting different histopathological subtypes of primary liver cancer. Front Oncol (2020) 10:1646. doi: 10.3389/fonc.2020.01646 33072550 PMC7543652

[34] Xiong L Chen H Tang X Chen B Jiang X Liu L. Ultrasound-based radiomics analysis for predicting disease-free survival of invasive breast cancer. Front Oncol (2021) 11:621993. doi: 10.3389/fonc.2021.621993 33996546 PMC8117589

[35] Jiao DC Qiao JH Zhu JJ Wang LN Ma YZ Lu ZD. [analysis of factors influencing the axillary lymph node metastasis and breast cancer-specific survival in patients with t1 breast cancer]. Zhonghua Yi Xue Za Zhi (2018) 98:3258–62. doi: 10.3760/cma.j.issn.0376-2491.2018.40.009 30392292

[36] Li Q Jiang T Zhang C Zhang Y Huang Z Zhou H. A nomogram based on clinical information, conventional ultrasound and radiomics improves prediction of malignant parotid gland lesions. Cancer Lett (2022) 527:107–14. doi: 10.1016/j.canlet.2021.12.015 34929334

[37] Wang S Chen Y Zhang H Liang Z Bu J. The value of predicting human epidermal growth factor receptor 2 status in adenocarcinoma of the esophagogastric junction on ct-based radiomics nomogram. Front Oncol (2021) 11:707686. doi: 10.3389/fonc.2021.707686 34722254 PMC8552039

[38] Tang J Tang J Jiang S Ma J Xi X Li H. Nomogram based on radiomics analysis of ultrasound images can improve preoperative braf mutation diagnosis for papillary thyroid microcarcinoma. Front Endocrinol (Lausanne) (2022) 13:915135. doi: 10.3389/fendo.2022.915135 36060960 PMC9437521

[39] Zhang D Wei Q Wu GG Zhang XY Lu WW Lv WZ. Preoperative prediction of microvascular invasion in patients with hepatocellular carcinoma based on radiomics nomogram using contrast-enhanced ultrasound. Front Oncol (2021) 11:709339. doi: 10.3389/fonc.2021.709339 34557410 PMC8453164

